# Edge effects reverse facilitation by a widespread foundation species

**DOI:** 10.1038/srep37573

**Published:** 2016-11-23

**Authors:** Laura J. Jurgens, Brian Gaylord

**Affiliations:** 1 Bodega Marine Laboratory and Department of Evolution and Ecology, University of California at Davis, Bodega Bay, California, United States of America

## Abstract

Dense aggregations of foundation species often mitigate environmental stresses for organisms living among them. Considerable work documents such benefits by comparing conditions inside versus outside these biogenic habitats. However, environmental gradients commonly arise across the extent of even single patches of habitat-forming species, including cases where stresses diverge between habitat interiors and edges. We ask here whether such edge effects could alter how habitat-forming species influence residents, potentially changing the strength or direction of interactions (i.e., from stress amelioration to exacerbation). We take as a model system the classic marine foundation species, *Mytilus californianus*, the California mussel. Results demonstrate that mussel beds both increase and decrease thermal stresses. Over a distance of 6 to 10 cm from the bed interior to its upper surface, peak temperatures climb from as much as 20 °C below to 5 °C above those of adjacent bedrock. This directional shift in temperature modification affects interactions with juvenile mussels, such that thermal stresses and associated mortality risk are higher at the bed surface, but substantially reduced deeper within the adult matrix. These findings provide a case example of how stress gradients generated across biogenic habitats can markedly alter ecological interactions even within a single habitat patch.

Many of the world’s most speciose ecosystems — including coral reefs, rainforests, seagrass meadows, and mussel beds — are dominated by high-biomass foundation species^1^ that can strongly influence environmental conditions (“habitat modification”^2^). Generally, these species are thought to facilitate, or have positive effects, on associated organisms by reducing abiotic stresses^3^,^4^. While stress reduction inside biogenic habitat patches is well documented^5^, studies also demonstrate how stands of habitat-formers can alter environmental conditions differently at habitat edges^8^,^9^. Environmental conditions can thus vary strongly across the spatial extent of a foundation species, leading to internal gradients that could rival in magnitude contrasts between within-stand locations and those outside. Moreover, if such internal stress gradients are strong enough, they could alter the strength or direction of habitat modification, even potentially changing the effect from one of stress reduction to stress exacerbation.

Within-patch variation in environmental conditions underpins many edge-effect findings, and research indicates that both terrestrial and marine foundation species can affect conditions differently depending on location within a habitat patch. For example, studies show increased temperature extremes at the periphery of forests but decreased variation towards the interiors^10^,^11^, depth-based shifts in how aquatic macrophytes influence dissolved oxygen and seawater pH^12^, and spatially variable effects of kelp beds on currents, with flow velocities declining in the center of beds but increasing along their margins^13^,^14^,^15^. Although these examples demonstrate qualitative spatial variation in the biophysical effects of habitat-formers, there have to our knowledge been no explicit investigations into how such within-patch variation might modulate the fundamental nature of facilitative interactions between foundation species and their residents.

We address this knowledge gap in a model system formed by a classic rocky shore foundation species, the California mussel, *Mytilus californianus*. California mussels create extensive three-dimensional beds, typically just a few centimeters deep, that host enormous taxonomic diversity spanning the three major groups of algae, 12 invertebrate phyla, fishes, and grasses, with up to 600 species in a region, and commonly more than 100 species in areas as small as 0.15 m^2 ^16,17,18. While environmental stresses are well studied for adult mussels forming these habitats^19^,^20^,^21^, conditions for habitat users are less understood. As with many other foundation species, environmental stresses are thought to be ameliorated for taxa resident within California mussel beds, including the high temperatures and desiccating conditions that commonly occur at low tide^7^,^17^,^18^,^22^,^23^,^24^,^25^.

Here, we ask whether edge effects in habitats formed by *M. californianus* could fundamentally alter the nature of facilitative interactions with their residents. We begin by quantifying cross-habitat differences in peak temperatures, contrasting mussel bed interior locations (predicted to remain comparatively cool) with upper bed surfaces (where mussels exposed to direct solar radiation at low tide heat quickly^19^,^26^). We compare thermal conditions in these two mussel bed locations to those on adjacent rock substrate, hypothesizing that bed interior locations will buffer temperatures more strongly than bed surfaces. We then ask whether such differences could affect the strength of facilitation by altering heat-driven mortality risk for a ubiquitous inhabitant of mussel beds: juvenile mussels. We chose juvenile mussels as model inhabitants because they occupy all layers of mussel beds across the species’ range, span body sizes common to the vast majority of other bed inhabitants, and, as they grow to adulthood, contribute to persistence of the habitat itself. To compare habitat effects at edges versus interiors, we use lethal thermal tolerance thresholds of juvenile mussels and the aforementioned temperature measurements to determine how much mussel bed surfaces and interiors either decrease or increase the frequency of juvenile exposure to lethal temperatures (relative to rock substrate). We also examine juvenile mussel survival of high-heat conditions in both bed surface and interior locations with a field outplant experiment. Since physiological tolerances and microhabitat preferences commonly vary by body size and life stage^27^,^28^,^29^, we ask also whether size-related differences in thermal tolerance or habitat use might shift the relative importance of facilitation by the mussel bed^30^. Through this linked series of experiments, we provide an empirical examination of how edge effects may alter the strength and/or existence of facilitative interactions inside assemblages of a widespread habitat-forming species.

## Results

### Fine-scale variation in thermal habitat modification

**Figure 1:**
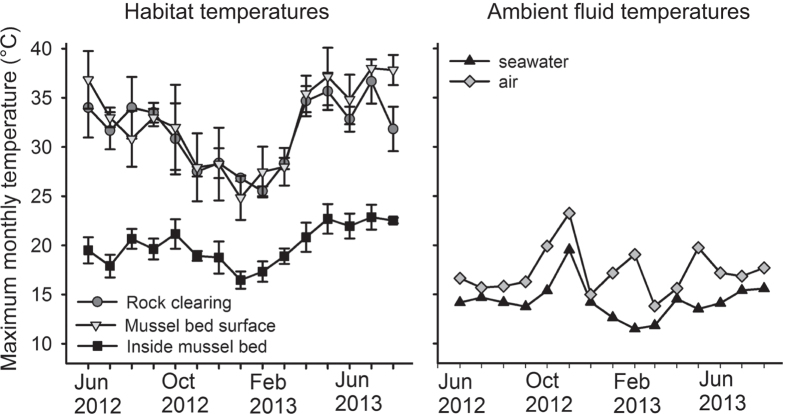
Monthly maximum temperatures by habitat location. Monthly maximum temperatures (±SD across replicate instruments) in exposed rock clearings and in two different habitats (the mussel bed surface and interior) modified by *M. californianus* over 15 months (left panel), along with temperature maxima recorded by sensors in adjacent seawater and air (right panel). All habitat data recorded at mid-intertidal elevations on horizontal surfaces at Bodega Marine Reserve, California, USA; sensor data from http://boon.ucdavis.edu.

**Figure 2:**
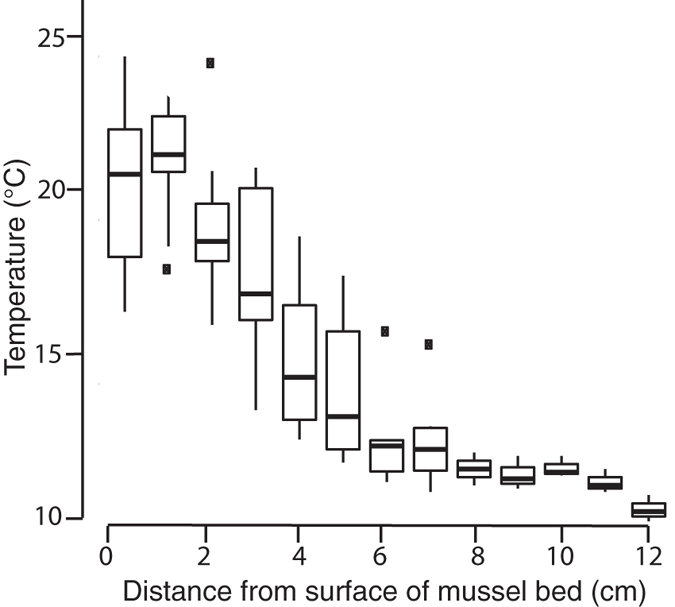
Vertical temperature gradient across mussel beds. Boxplots of temperature measurements by depth within mussel beds (N = 4) at Bodega Marine Reserve, California, USA. Data are from measurements taken within 30 minutes on a single day (18 April 2013; ambient air temperature: 11 °C) at the same shore elevation (2.1 m above MLLW).

Our data indicate that peak temperatures at low tide can differ substantially between interiors and upper surfaces of mussel aggregations (Fig. 1), and that these locations mark the end points of a strong, within-habitat gradient in thermal maxima (Fig. 2). Relative to adjacent bedrock, mussel bed microhabitats simultaneously elevated and reduced peak temperatures, depending on location. Interior mussel bed microhabitats remained below 25 °C even on the hottest days (Fig. 1), and peak values were reduced by an average of 10 to 15 °C compared to adjacent bedrock. In contrast, and particularly in months of high solar radiation between late spring and fall, monthly thermal maxima on mussel bed surfaces exceeded bedrock maxima by 2 to 5 °C (Fig. 1), occasionally reaching temperatures above 40 °C. Thermal maxima inside mussel beds were consistently lower, and less variable, than at mussel bed surfaces 6 to 10 cm away, or in adjacent rock clearings (see also Supplementary Information online, Fig. S1, for a sample time series). Temperatures recorded within and on the surface of mussel beds differed by up to 19.3 °C over the course of a month and 20.7 °C over a single day.

Monthly temperature maxima within the three microhabitats were not well approximated by temperatures measured in surrounding air and seawater, and also exhibited different patterns of seasonal variation (Fig. 2). Ambient air and water temperatures remained below 20 °C except during November 2012, when air and seawater temperatures peaked at 23 °C and 19.5 °C, respectively (Fig. 2; see also Supplementary Information online, Fig. S2, for correlation plots).

### Consequences for intraspecific interactions with juvenile mussels

**Figure 3:**
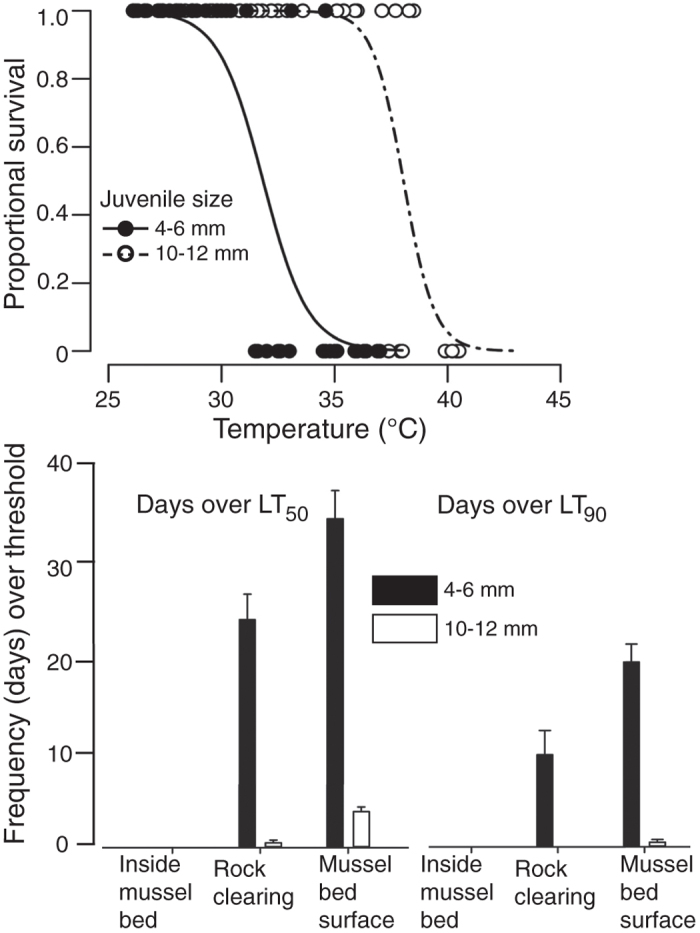
Thermal tolerances, and frequency of exceeding them by habitat, for two size classes of juvenile mussels. (**A**) Survivorship curves for two size classes of juvenile mussels as a function of aerial temperature, based on 4-hour laboratory challenge experiments (relative humidity 65 ± 1%). (**B**) Number of days (±SE) that habitat temperatures exceeded moderate (LT_50_, left) and severe (LT_90_, right) lethal thresholds for small (4–6 mm) and large (10–12 mm) juvenile mussels inside mussel beds, in rock clearings, and at mussel bed surfaces.

Results of laboratory thermal trials on juvenile mussels indicate that the thermal gradient we found across mussel beds crosses physiological thresholds, and therefore different locations within the bed affect juvenile mussel mortality risk in different ways. We also found that the consequences of this gradient are likely to vary by juvenile size. When tested in air (the relevant condition for such high temperatures, which occur at low tide in this system), body size strongly affected lethal thermal tolerances of juvenile mussels, as expected from prior thermal studies of ectotherms and biomechanical principles^19^,^31^,^32^. Mussels 4 to 6 mm in body length exhibited lower aerial thermal tolerances (as measured by lethal thresholds quantified as the temperature at which fifty percent mortality was observed “LT50”; see *Methods* for further details) compared to larger (10–12 mm) juveniles (Fig. 3A; LT_50_ = 31.8 versus 38.1 °C; LT_90_ = 34.0 versus 39.7 °C for small and large size classes, respectively; multiple logistic regression (MLR), null: χ^2^ = 97.7, degrees of freedom (df) = 79; temperature: χ^2^ = 32.9, df = 1, P < 0.001; size: χ^2^ = 34.4, df = 1, P < 0.001; interaction: χ^2^ = 0.4, non-significant: P = 0.53).We also conducted trials in seawater for comparison, and found no size dependence of thermal tolerance (LT_50_ and LT_90_ ~ 37 °C for both size classes; see also Supplementary Information online, Fig. S3), suggesting that effects of body size on thermal tolerance at low tide, at least for juveniles in these size ranges, may derive primarily from consequences of heat-exacerbated desiccation.

These tolerance data combined with our field measurements of thermal maxima indicate that interior mussel bed habitats had positive, while bed surface habitats had negative, effects on juvenile mussel exposure to high temperatures, relative to rock surfaces (Fig. 3). The strength of both effects, as measured by the frequency of exposure to temperatures over lethal limits (see *Methods*), reflected size-based differences in thermal tolerance. Estimated exposure of small juvenile mussels to lethal temperatures depended on location within or on the mussel bed (Fig. 3B; LT_50_: H = 9.64, df = 2, P < 0.001; LT_90_: H = 9.75, df = 2, P < 0.001; see also Supplementary Information online, Fig. S4). Mussel bed surfaces regularly exhibited temperatures beyond the lethal tolerances of small juveniles, even more so than rock clearings, while mussel bed interiors strongly attenuated thermal extremes (Fig. 3B, P < 0.05 for all pairwise microhabitat comparisons). Differences in thermal risk among microhabitats did not depend strongly on the mortality threshold considered for small juveniles (LT_50_ and LT_90_ in Fig. 3B). In bed surface habitats, temperatures occasionally exceeded even the more robust LT_50_ (38 °C) of large juvenile mussels (Fig. 3; LT_50(freq)_:H = 9.19, df = 2, P = 0.005, LT_50(hrs)_:H = 9.37, df = 2, *P* = 0.01; P < 0.05 between surface and other habitats only). Lethal risk for larger juveniles in rock clearings did not differ significantly from the corresponding risk in mussel bed interiors, despite overall higher temperatures in rock clearings. Temperatures greater than the LT_90_ (~40 °C) of large juveniles were sufficiently rare that we did not detect a difference in their occurrence as a function of habitat (H = 4.4, df = 2, P = 0.45).

**Figure 4:**
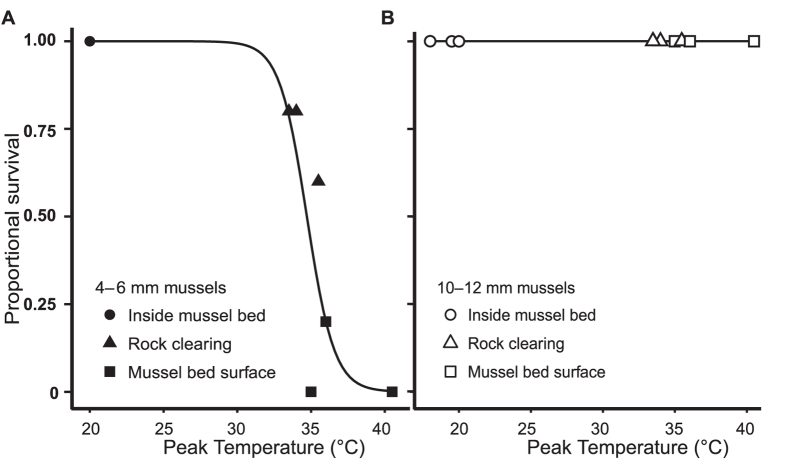
Field survivorship of juvenile mussels by microhabitat on a high heat day. Results of *in situ* field exposures of (**A**) small and (**B**) large size classes of juvenile mussels in two habitats modified by mussel beds (surface and interior), relative to rock clearings, with fitted logistic functions (N per size class = 90).

Results of a field outplant experiment substantiated the expected size- and habitat-dependent differences in vulnerability of juvenile mussels. We found zero mortality of either size class inside mussel beds on a high-heat day (Fig. 4). Small juveniles, however, exhibited appreciable mortality on mussel bed surfaces (Fig. 4; mean proportional mortality: 0.93, SD: 0.11). These mortality rates exceeded those of mussels in adjacent rock clearings (mean proportional mortality: 0.27, SD: 0.11). All individuals of the larger size class survived regardless of microhabitat (Fig. 4).

**Figure 5:**
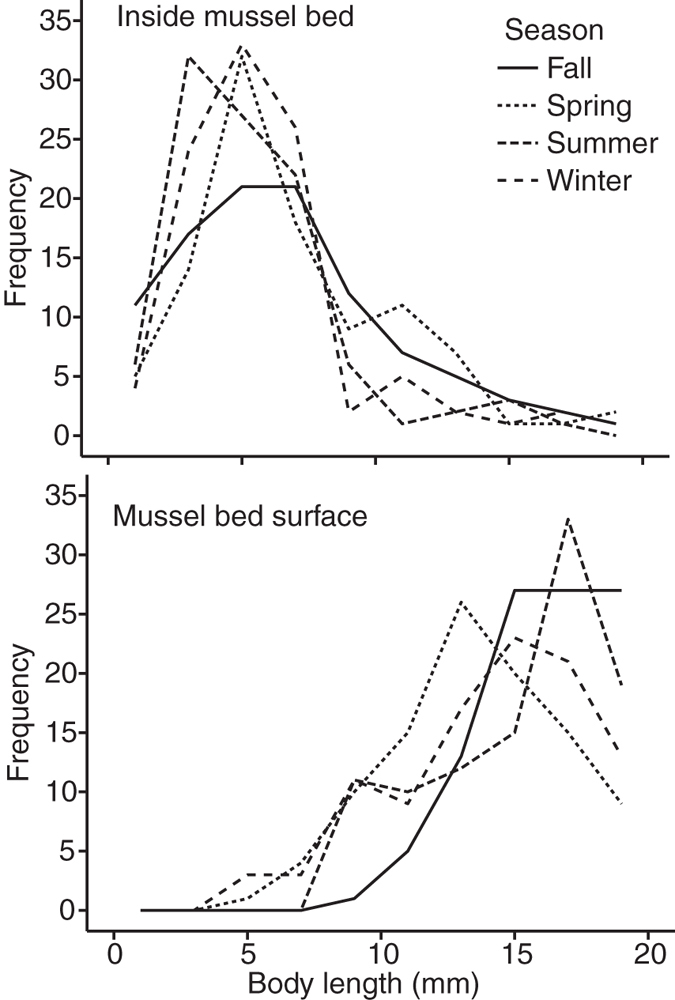
Juvenile mussel size distributions at the surface and interior of mussel beds. Frequency of juvenile mussels by size class as found in the field in the interior of mussel beds (upper panel) and in mussel bed surface microhabitats (lower panel), by season (2 mm size bins, N = 100 individuals per season).

Size distributions of juveniles living naturally in the field differed consistently between mussel bed interiors and surfaces, across all seasons (Fig. 5), suggesting different size classes may be exposed to different thermal regimes across the habitat. Small juvenile mussels were concentrated inside the bed, including 100% of sampled individuals under 3 mm in length. Larger sizes were more common at the mussel bed surface, and we observed a marked shift in size frequency between the two mussel bed microhabitats at about 8 mm in body length (Fig. 5).

## Discussion

Our results demonstrate that differences in how foundation species modify physical conditions at habitat edges versus interior locations can cause environmental stressors to vary as strongly within a habitat patch as they do between locations inside and outside it. Consequently, environmental gradients within aggregations of habitat-forming species can be steep enough to drive both mitigation and exacerbation of the same stressor, and can do so simultaneously in closely adjacent portions of a habitat patch. This phenomenon differs from most classic representations of habitat modification that emphasize only stress-reducing benefits of foundation species. Findings here also complement theories of spatial variation in facilitation operating at much larger spatial scales (e.g., the Stress Gradient Hypothesis^2^,^30^) by highlighting the importance of small-scale, within-patch variation in the strength and existence of facilitative interactions.

This study documents, in particular, peak temperatures inside mussel beds that were reduced by up to 20 °C, while bed surface temperatures at locations less than 10 cm away were elevated by up to 5 °C, relative to nearby rock surfaces. The extent of these differences exceeds variation in temperature maxima reported across 14° of latitude for solitary adult mussels (14 °C)^33^. Mechanistically, it appears that microhabitats within the bed are buffered from solar-driven warming by shading and relatively inefficient heat conduction from the surface to the interior, potentially driven by the tendency for adults in the matrix to touch each other only over small portions of their shells^26^. In contrast, the direct impingement of solar radiation on dark shells of mussels at the bed surface results in more extreme warming (Fig. 1).

The strong thermal gradient we found within mussel beds appears to drive marked differences in mortality risk for juvenile mussels, spanning conditions from well below to above lethal thermal tolerances. These effects represent underappreciated impacts on not only the strength but also the existence of intraspecific facilitation within this classic foundation species. Our results indicate that small juvenile mussels are particularly vulnerable to heat-driven mortality, and therefore a critical agent of long-term persistence for *Mytilus californianus* may be increased survival of vulnerable early juveniles that settle within the heat-buffered bed matrix (relative to rock or mussel bed surface habitats). This pattern appears to differ from the situation in many foundation species, where adults frequently inhibit the recruitment of early life stages of their own species (e.g., forest trees, kelps, and seagrasses^34^,^35^,^36^,^37^).

At the same time, results here demonstrate that stress gradients within biogenic habitats may affect habitat users in ways that depend strongly on physiological tolerance. We found very few mussels less than 8 mm in body length at bed surfaces, but larger, more heat-tolerant juveniles disproportionately occupied these high-stress regions (Fig. 5). This size-dependent habitat partitioning (i.e., smaller juveniles within the bed; larger ones more often at the surface) may arise through several mechanisms, including differences in predation, food availability, or water flow (hypothesized to decrease retention of small recruits at bed surfaces for the mussel *Perumytilus purpuratus* in Chile^38^), as well as the size-dependent differences in thermal vulnerability we quantify here. Severe, thermally driven mortality of small size classes at the bed surface could act to remove settlers, or impose selection over time against larval settlement in such high-stress locations. Larvae settle year-round in this region^39^, and monthly maximum temperatures between November and February at the bed surface were generally within the range of thermal tolerance of the small juveniles we tested (Figs 1 and 3). If larvae commonly settled onto the surface of the bed and were then killed by high temperatures, we would have expected to find more juveniles under 6 mm in body length at the bed surface in the winter before temperatures rose later in the year. That we did not find such a cohort (Fig. 5) suggests that either new settlers were killed rapidly by other factors or driven to relocate before we could detect them, or that they preferentially settled inside the bed directly. Indeed, *M. californianus* and other mussel species appear to prefer filamentous substrates such as algae or byssal threads (more abundant deeper in the bed) to the adult shell surfaces preferred by later stages^40^,^41^, but the proximate and ultimate causes of these patterns remain open questions. Certainly juvenile mussels are capable of movement, and are known to relocate after initial settlement^42^,^43^,^44^, so habitat partitioning could arise if juveniles of different sizes move among preferred microhabitats.

Correlations between edge-driven environmental gradients and inhabitant organism distributions have been documented in a variety of systems and taxa, including lichens and amphibians in forests^45^,^46^, and even enhanced-stress regions of such gradients could be exploited by inhabitants capable of tolerating conditions there. In addition to providing extensive surface area for colonization, advantages of high-heat locations at the bed surface may emerge for large juvenile mussels and other heat-tolerant species. These include increased flow^47^, and thus enhanced delivery of planktonic food, as well as reductions in negative interactions with more heat-sensitive species, including interactions driven by overgrowth or predation^48^,^49^. These latter situations provide examples of how indirect effects of within-patch stress gradients could influence species interactions by separating or concentrating resident taxa along axes of physiological tolerance. They also underscore the potential for future increases in high-heat events, for example due to global climate change, to alter the strength of any indirect interactions that rely on thermal effects of the habitat forming species (e.g., temperature-driven changes in predator consumption rates). While larger juveniles appear capable of tolerating most high-temperature conditions, they also appear to be living very close to their thermal tolerance limits at the bed surface (e.g., Fig. 3) and could in fact become a demographic weak link if peak temperatures rise in coming decades.

The type of within-patch stress gradients we found here could work similarly to alter the strength or existence of facilitation in a range of systems. Edge-driven variation, and even directional shifts, in the biophysical or biochemical effects of habitat-formers on environmental stressors are known in a variety of terrestrial and aquatic habitat-forming species. For example, wind speeds vary adjacent to and within forests — a phenomenon well established in studies of edge effects^10^,^11^ — and these patterns are predicted to facilitate or inhibit the dispersal of plants and insects, depending on their proximity to the forest edge^50^. Similarly, mangrove stands may differentially affect the incidence of flow-driven disturbance for root-colonizing organisms depending on whether these organisms live near windward edges of stands or within the dense interior^51^. Biochemical gradients could similarly influence interactions between residents and habitat-formers in stands of aquatic vegetation, which can create divergent exposures to low pH and dissolved oxygen across depth, as respiration outpaces photosynthesis in deeper waters^12^. These gradients could increase physiological stress for benthic fish and invertebrates, while the reverse may periodically relieve stresses for organisms near the water surface. Considered in light of results presented here, these examples suggest that within-patch stress gradients could warrant further attention as potentially common modifiers of facilitative interactions. In particular, assumptions of simple stress reduction (i.e., “habitat amelioration”) based on measurements in single or similar locations may inadequately characterize facilitative interactions across the full domain of a given habitat-forming species. Net positive effects of habitat-formers could be most common, and are often of critical importance to facilitated populations. However, predicting when, where, and the degree to which facilitation occurs may in many cases require explicit consideration of within-patch stress gradients, coupled with an understanding of the physiological thresholds of potentially facilitated species and/or life stages.

**Figure 6:**
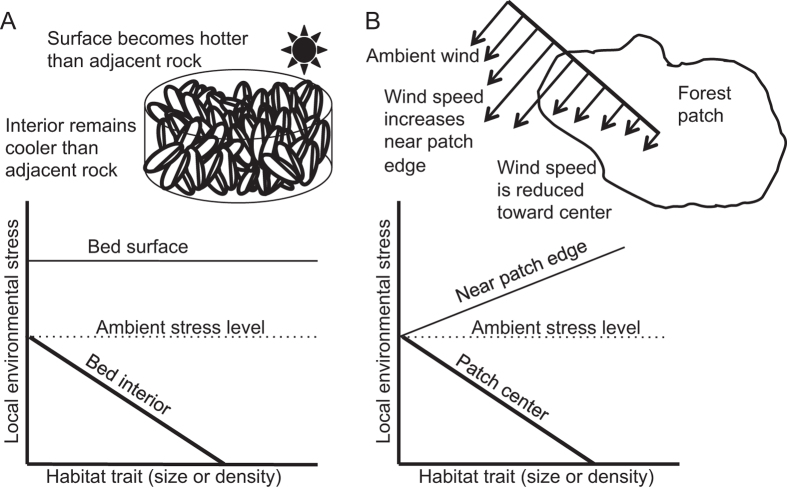
Incorporating edge effects into conceptual models of facilitation. Conceptual model depicting how edge effects could drive spatial heterogeneity in habitat modification within the domain of a habitat-former. The heavy black lines indicate, for each of two habitat types, a previously described pattern in which increased habitat density or size of an aggregation cause greater amelioration of environmental stress^59^. Dotted lines indicate ambient stress levels outside the biogenic habitat. As explored here, habitat modification could exacerbate environmental stresses as well as mitigate them (thin solid lines). (**A**) Case of a vertical core through a mussel bed in which interior locations experience reduced temperatures via shading and poor heat conduction, while the bed surface encounters elevated temperatures compared to nearby bedrock, as small, dark shells heat rapidly under solar radiation. In this first scenario, only interior reductions in temperature will likely depend on the density or size of the mussel bed. (**B**) An alternative example in which a biogenic habitat again ameliorates and exacerbates environmental stress simultaneously, but both processes depend on habitat density or size. Here, the tendency for a stand of forest trees to slow wind speeds in its interior increases with the density or spatial extent of the stand. Likewise, the tendency for the forest stand to divert winds around it and therefore induce flow acceleration along its edges rises as the density and/or dimensions of the stand increases.

Although such issues do not yet appear in most conceptual considerations of facilitation and its consequences, findings here suggest that incorporating within-patch stress gradients into ecological theory could lead to a richer understanding of organismal interactions. Facilitation by habitat modification is often conceived as driving monotonic decreases of environmental stresses within the spatial extent of a biogenic habitat, with such declines becoming more marked as the density or size of the habitat-former increases. By including the possibility of within-patch shifts in habitat modification, we can envision a broader set of spatially dependent interactions between habitat-users and habitat-formers (e.g., Fig. 6). Such details could be important for predicting population responses to a shifting climate in the many cases where habitat-formers have strong local effects on climate-related parameters such as temperature, humidity, precipitation, and water chemistry. Here, we found that quantifying a within-patch gradient in thermal stress enabled a clearer understanding of intraspecific relationships driving juvenile mortality risk in a dominant foundation species. Taken together, these findings underscore the benefits of including the sometimes dramatic and opposing patterns of habitat modification into efforts to identify relationships among, and vulnerabilities of, the numerous taxa associated with habitat-forming species.

## Methods

### Study location and organisms

We conducted field portions of the study at the Bodega Marine Reserve (BMR; 38°19.1′N, 123°4.4′W) in Bodega Bay, California, USA on horizontal rock benches at a constant mid-intertidal shore elevation, 2.1 m above mean lower low water (MLLW) that supported healthy mussel beds 6 to 10 cm thick. There is substantial wave action in the region and the study location is directly exposed to breaking waves. California mussels broadcast spawn throughout the year, with the peak season varying geographically^52^,^53^. Larvae develop in the plankton, then settle and metamorphose into juveniles that occupy adult habitat on the shore, with settlement heavy in mussel beds, but also occurring on barnacles and seaweeds^54^. Our experiments focused on post-settlement juveniles spanning sizes 1–20 mm in body length, characteristic of early recruits to individuals approaching reproductive age (at ~25 mm^53^), and representing sizes typical of individuals of many other species that reside within mussel beds.

### Fine-scale variation in thermal habitat modification

To assess spatial differences in peak temperature patterns within mussel bed habitats, and relative to adjacent bedrock, we measured peak temperatures at the mussel bed surface (where incident solar radiation is strongest), in the deepest locations inside the bed near the rock substrate, and in rock clearings nearby (within 30 cm) that we created by scraping 30-cm diameter circles of bedrock clear of epibiota. We recorded temperatures every 30 minutes for 15 months between June 1, 2012 and September 1, 2013 with small loggers fixed to horizontal surfaces in each microhabitat with marine epoxy (DS-1921-G thermochron iButtons, Maxim®, San Jose, CA, USA; N = 12; 4 per microhabitat; calibrated, waterproofed in Parafilm®, and coated with 5 g neutral-colored Zspar® SplashZone marine epoxy). This approach allowed us to capture a full year of data bracketed by two summers, in which we expected a higher probability of stressful heat events^19^,^20^,^55^. We maintained a continuous deployment by exchanging loggers that approached full data capacity every 3 to 5 weeks, depending on tidal access.

We also examined the nature of the full thermal gradient by measuring temperatures as a function of multiple vertical positions in the bed (single measurements every 1 cm within four replicate mussel beds) using a high-resolution thermocouple thermometer (Fluke® 52-II, Everett, WA, USA; equilibration time 2 to 4 s). We accessed accompanying air and seawater temperature data from sensors located within 100 m of the study location^56^, in order to relate surrounding physical conditions to those arising within our focal microhabitats (see Supplemental Information online for detailed results).

### Consequences for intraspecific interactions with juvenile mussels

We used linked field and laboratory experiments to examine how microhabitat differences among mussel bed interiors and surfaces, relative to rock clearings, influenced the exposure of juvenile mussels to mortality-inducing high temperatures. We considered mussel body size in these efforts. Size is a well established factor affecting the thermal tolerances of invertebrates, since differences in ratios of surface area to volume across size can strongly influence heat gain and water loss, the latter of which can also feed back to affect organism temperature^31^,^32^. We first measured lethal thermal tolerances of two size classes of sub-adult mussels, 4 to 6 mm and 10 to 12 mm in body length, in the laboratory, then compared the number of days and cumulative time for which temperatures in the field exceeded these lethal thresholds within the three microhabitats (mussel bed interior, bed surface, adjacent rock). Next, we replicated field-based trials until we captured an extreme high-temperature day, and assessed juvenile survivorship as a function of size and microhabitat *in situ*. For laboratory and experimental outplant components of the project, we sourced all animals from mussel beds at BMR and held them in common conditions at Bodega Marine Laboratory (BML) adjacent to the field site, in filtered, flowing seawater at ambient temperature, 11 to 13 °C. Lastly, we measured size distributions of juveniles living in the interiors or on the surfaces of mussel beds in the field to examine how potential size-based differences in habitat use may affect the relative exposure of juveniles to potentially divergent thermal conditions. Further details appear below.

### Size-specific thermal tolerance assays

In intertidal regions occupied by *M. californianus*, ocean temperatures remain cool relative to those that can occur during low-tide emersion. Hence, the most severe temperature stress is unavoidably linked with desiccation, which increases under warmer conditions. We therefore conducted thermal tolerance experiments in air to simulate realistic low tide conditions, under 65 ± 1% relative humidity (RH), which was the mean of our measurements in exposed microhabitats at BMR, measured 1 m above the substrate on 12 sunny days in 2012 with a Kestrel® 4500 Portable Weather Station. We repeated thermal tolerance trials in seawater (i.e., without heat-exacerbated desiccation) for comparison. To implement thermal trials over durations typical of low-tide exposure, we used for each a 1.5-hour ramp-up time from ambient seawater temperature followed by a 4-hour treatment at peak temperature, and a 24-hour recovery period in running seawater. Following similar procedures to other researchers^57^, we established a temperature gradient using an aluminium bar with one end connected to a heat source and another to a cooling water bath, with holes drilled along its length to fit 2 mL microcentrifuge tubes, each containing a single mussel. We randomly allocated four mussels of the same size class to each of 10 rows in the heat bar (within-row temperatures were ± 0.1 °C). After the ramp up time, rows reached peak temperatures between 26 °C to 42 °C, with row-to-row differences in increments of ~1.2 °C (N = 40 mussels per size class per fluid). For each fluid (air and seawater), both size classes were tested simultaneously to avoid potential differences in treatment conditions. We removed lids from microcentrifuge tubes to allow desiccation during aerial trials, and reduce the likelihood of hypoxia during seawater trials, in which we also monitored water levels to ensure minimal evaporation and associated changes in salinity. After the 24-hour recovery period, we tested individuals for vitality using foot movement or, if none was apparent, examined each under a dissecting microscope for mantle tissue response to prodding and siphon function. We found 7% mortality of 24-hour survivors at 96 hours post-trial. However, we used only the 24-hour post-trial data for calculating acute lethal temperature tolerances, since subsequent mortality may have been influenced by other factors.

After the trials, we determined both the temperature that led to 50% mortality (LT_50_) in the sample population of each size class (a metric of substantial risk), as well as the 90% mortality threshold (LT_90_) for each size class (representing a temperature capable of inducing severe demographic consequences). We calculated these lethal thresholds by fitting the survivorship data with a generalized linear model (binomial distribution: individuals survived or not; logit link) and back-calculating the median lethal temperature “dose” that drove 50% (LT_50_) and 90% (LT_90_) mortality for each size class in R (version 3.1.2, R Development Core Team) with the MASS package^58^. We also tested the hypothesis that size class affected lethal thermal tolerance via multiple logistic regression (MLR) in R (again, due to binary data). We report deviance-based statistics (χ^2^). See Supplemental Information online for R code.

We then compared the number of days in which temperatures in each of the three microhabitats (mussel bed surfaces, mussel bed interiors, and exposed rock surfaces) exceeded the laboratory-determined, LT_50_ and LT_90_ values of each size class (from tests in air, since such temperatures occur at low tide) over the 15-month temperature logger deployments. We chose a simple nonparametric approach for data analysis due to the temporal components of the habitat temperature data, and because habitat temperatures never exceeded lethal thresholds inside mussel beds. We analysed the number of days temperatures exceeded lethal threshold by habitat based on relative ranks with Kruskal-Wallis tests and pairwise post-hoc comparisons (Student-Neuman-Keuls “SNK” procedure), and did so separately for each size class, given their different thermal tolerances.

### Field tests of juvenile mussel survival

We used an outplant experiment to determine whether mortality patterns under field conditions on hot days aligned with expectations based on the above comparisons of field-measured microhabitat temperatures and laboratory-measured thermal tolerances. Given the unpredictability of extreme temperature events in nature, we undertook the outplants on multiple days that experienced combinations of bright sun, low wind, and low tide near solar noon that could lead to hot intertidal conditions. On each of five such days between April and June 2013, we placed sets of individuals of both size classes in each of the three focal microhabitats for three hours (N = 90 per size class per trial). The three-hour duration of field exposure represents the conservative end of low-tide exposure times at BMR, which can often exceed six hours. We collected experimental animals from BMR three days prior to each experiment from inside mussel beds to reduce the likelihood of differential acclimatization by size class prior to the experiment. At the time of the trial, we verified vitality using foot movement, and allocated individuals of each size class randomly to three experimental beds formed of 30 adult mussels (40 to 60 mm in body length, placed in a dense formation 7 cm deep to mimic adjacent natural beds). We monitored temperatures every 30 minutes, provided a 24-hour recovery, and assessed post-trial vitality as described above for laboratory assays. We avoided the use of adhesives, which could alter experimental outcomes, and instead simply placed juveniles on adult shell or rock surfaces manually, with each juvenile location marked by a 1 cm strip of brightly colored rubber located at least 5 body lengths away. One of the five outplant days (May 4, 2013) yielded temperatures over 30 °C (the minimum temperature at which we observed juvenile mortality in laboratory experiments) in at least one of the focal habitats, and we therefore centered our attention on results from this date as informative for understanding potential consequences of mussels’ exposure to extreme high-temperature events. Figure 4 presents these data graphically. Consistent with expectations, no juveniles died on the other, cooler outplant dates.

### Size-specific habitat distributions

We examined the potential for size-based differences in juvenile mussel microhabitat use within the adult matrix, since any such patterns coupled with the hypothesized within-bed thermal gradient could help predict post-settlement size classes most vulnerable to heat-driven mortality. We measured, once in each of four seasons at BML, the body lengths of the first 100 sub-adult mussels we encountered, either on mussel bed surfaces or inside beds, within 5 to 10 (depending on the number of juveniles encountered) randomly placed 0.25 × 0.25 m quadrats. We examined beds at shore elevations ranging from 1.7 to 2.5 m above MLLW. We first examined the surface with a hand lens, measured individuals encountered, and then removed adult mussels and repeated this procedure in the interior, in each quadrat. Due to the presence of too few juveniles on rock surfaces, we did not include the latter habitat in our comparisons. We analyzed the log-transformed data of body length using a factorial ANOVA with mussel bed location and season as predictors.

## Additional Information

**How to cite this article**: Jurgens, L. J. and Gaylord, B. Edge effects reverse facilitation by a widespread foundation species. *Sci. Rep.* **6**, 37573; doi: 10.1038/srep37573 (2016).

**Publisher's note:** Springer Nature remains neutral with regard to jurisdictional claims in published maps and institutional affiliations.

## Supplementary Material

Supplementary Information
