# Supplementary material for: Edge effects reverse facilitation by a widespread foundation species
Source: Sci Rep. 2016 Nov 23;6:37573. doi: 10.1038/srep37573 (PMC5120328; doi:10.1038/srep37573)
Supplement: Supplementary Information [file srep37573-s1.pdf]

Edge effects reverse facilitation by a widespread foundation species

Jurgens, L. J and B. Gaylord

Supplementary Information

1. Methods Supplement

Temperature logger calibration, coating and attachment

Before deployment, we calibrated the iButton loggers in an ice bath, and encased them in Parafilm® and 5 g of neutral-colored marine epoxy (Zspar® SplashZone A-788 compound). We then affixed them in each microhabitat using the same epoxy. We deployed iButtons at the bed surface by attaching them to horizontally oriented single adult mussel shells. We placed loggers inside the bed by pulling back small patches of mussels, scraping and rinsing the substrate of sediment, affixing the instrument, and re-attaching the mussels using small amounts of marine epoxy, which we also used to mark the location for later instrument retrieval. We affixed loggers in rock clearings (30 cm diameter to eliminate shading of instruments by nearby organisms) directly to scraped and rinsed bedrock with the marine epoxy and maintained the area clear of epiphytes or epifauna during the deployment.

We tested the accuracy of the iButtons on rock and mussel surfaces in natural sunlight with and without the coating treatment (film and epoxy) using a high-resolution thermocouple thermometer (Fluke® model 50-II). Measurements by iButtons encased in film and epoxy logged temperatures within 0.5° C of the thermometer measurements, matching the reported precision of the instruments. This treatment led to more accurate temperature measurements than bare iButtons (which are metal and tended to overestimate substrate temperatures when exposed to sunlight). We maintained a continuous deployment by exchanging loggers that had reached full data capacity every 3 to 5 weeks, depending on tidal access.

Lethal temperature trials for juvenile mussels

We measured the temperature of each mussel shell surface every half hour during the treatment using a high-resolution thermocouple thermometer. Our use of a 1.5-hour ramp-up time and 4-hour treatment at maximum temperatures simulated low tide exposure. Mussels were then plunged in ambient seawater, as would occur with a returning tide. After 24 hours of recovery in running seawater, we tested individuals for vitality using foot movement or, if none was apparent, examined each under a dissecting microscope for mantle tissue response to prodding.

R Code for analyses of juvenile mussel thermal tolerance

```
#R Code for calculating lethal thresholds of juvenile mussels
##code adapted from http://lukemiller.org/index.php/2010/02/calculating-lt50-median-lethal-
temperature-aka-ld50-quickly-in-r/
data=read.csv("HeatBlk_Sm_Air.csv")
attach(data)
#create array of response variables
model.results=glm(LIVE~Peak_temp,binomial)
summary(model.results)
#use MASS library function dose.p to calculate median lethal dose of factor
##note that p here is SURVIVAL proportion, so p=0.1 is the dose that caused 90% mortality
library(MASS)
dose.p(model.results,p=c(0.5,0.1))

#Multiple logistic regression for mussel heat block data
data<- read.csv("MLR_MusselData.csv")
#Analysis for experiment in air
Temp<-as.factor(data$Peak)
Fluid<-as.factor(data$Environment)
fit <- glm(Dead ~ Temp * Size, data=subset(data, Fluid=="AIR"), family='binomial')
```

```
summary(fit)

# Does "Type I" tests, where the terms are added sequentially to the model:

anova(fit, test="Chi")

#Analysis for experiment in seawater

fit <- glm(Dead ~ Temp*Size , data=subset(data, Fluid=="Water"), family='binomial')

summary(fit)

Water<- subset(data,Fluid=="Water")

anova(fit, test="Chi")
```

2. Results Supplement

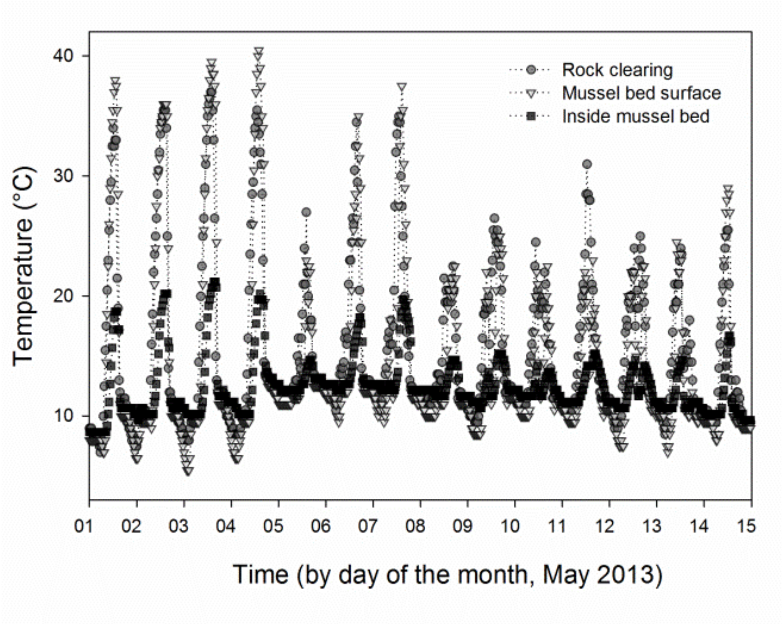

Figure S1. Time series of microhabitat temperatures. Habitat temperatures, measured every 30 minutes during the first two weeks in May 2013, show variation by microhabitat and with tidal cycle.

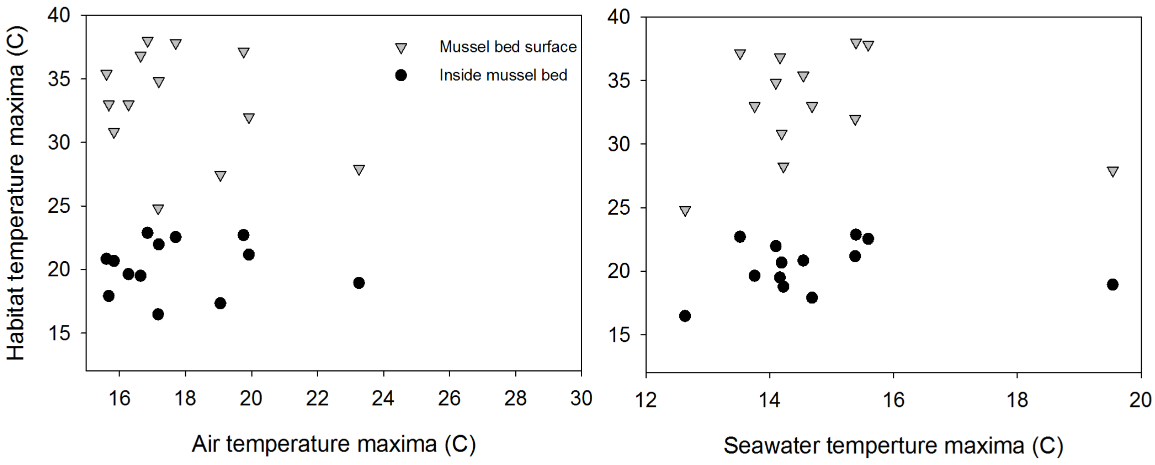

Figure S2. Correlation plots of habitat temperatures with ambient air (left) and water temperatures (right). No correlation is evident.

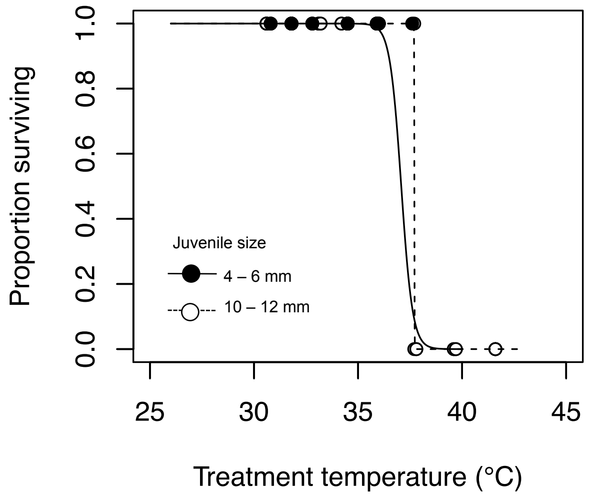

Figure S3. Lethal thermal tolerances for juvenile mussels of two size classes in seawater. Tolerances did not differ by size in seawater (*MLR:* null $\chi^2 = 76.0$, df = 79; temperature: $\chi^2 = 64.7$, df = 1, $P < 0.001$; size: $\chi^2 = 0.8$, non-significant: $P = 0.4$; interaction: $\chi^2 = 1.4$, non-significant: $P = 0.2$).

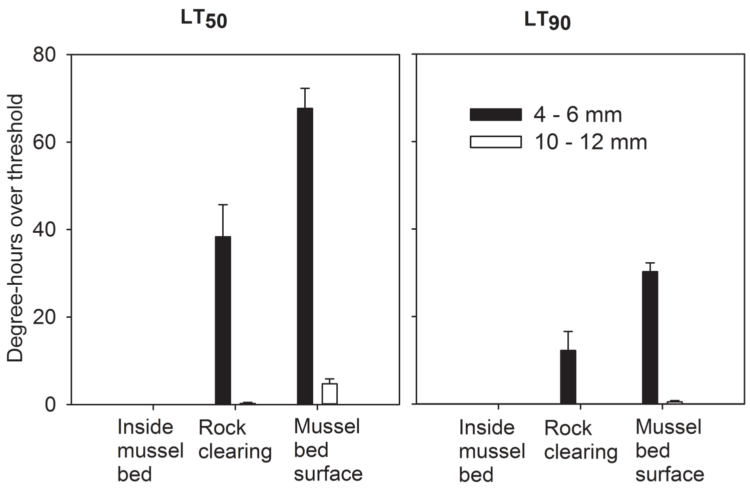

Figure S4. Cumulative duration of exposure to lethal temperatures by microhabitat. Patterns of chronic exposure to high temperatures by microhabitat, for two size classes of juvenile mussels, depicted as cumulative duration in hours that temperatures exceeded lethal tolerances (LT_{50}, left and LT_{90}, right; LT_{50}: H = 10.2, df = 2, $P < 0.001$; LT_{90}: H = 10.2, df = 2, $P < 0.001$). Patterns here are nearly identical to those for the more acute metric, the number of days in which temperatures exceeded these lethal thresholds, depicted in Fig. 4.
